# RAD18 confers radioresistance of esophagus squamous cell carcinoma through regulating p‐DNA‐PKcs

**DOI:** 10.1002/cam4.4754

**Published:** 2022-04-14

**Authors:** Xiaoqing Li, Shitao Zou, Liangsu Zhou, Aidi Gao, Jing Xu, Chao He, Jundong Zhou, Shuhua Wu, Yihong Chen

**Affiliations:** ^1^ Suzhou Cancer Center Core Laboratory The Affiliated Suzhou Hospital of Nanjing Medical University Suzhou Jiangsu China; ^2^ Department of Radiation Oncology The Affiliated Suzhou Hospital of Nanjing Medical University Suzhou Jiangsu China; ^3^ Department of Neurology The Second Affiliated Hospital of Nanjing Medical University Nanjing China; ^4^ Department of Geriatrics The Second Affiliated Hospital of Soochow University Suzhou Jiangsu China; ^5^ Department of Radiation The First Affiliated Hospital of Wanna Medical College Wuhu Anhui China

**Keywords:** esophageal squamous cell carcinoma, NHEJ, p‐DNA‐PKcs, RAD18, radioresistance

## Abstract

**Background:**

Radiotherapy has recently become more common for the treatment of esophageal squamous cell carcinoma (ESCC). Radioresistance, on the other hand, continues to be a major issue because it interferes with the effectiveness of ESCC radiation. It has been demonstrated that RAD18, an E3 ubiquitin‐protein ligase that regulates translesion DNA synthesis (TLS), is implicated in the regulation of genomic integrity and DNA damage response.

**Methods:**

In the present study, immunohistochemical staining and western blotting were utilized to determine RAD18 expression in ESCC tissues and cells. ESCC cell proliferation was determined using a colony formation assay. Immunofluorescence staining, comet assay, and homologous recombination (HR)/non‐homologous end‐joining (NHEJ) assays were conducted to examine the effect of RAD18 on the DNA damage response in ESCC cells.

**Results:**

We found that high RAD18 expression was positively associated with a poorer prognosis in patients with ESCC who received radiotherapy. Downregulation of RAD18 expression significantly increased the sensitivity of ESCC cells to irradiation. Moreover, RAD18 knockdown prolonged the repair kinetics of γH2AX foci and resulted in longer comet tails. Furthermore, loss of RAD18 expression markedly decreased non‐homologous end‐joining (NHEJ) activity, but it did not affect homologous recombination (HR)‐mediated double‐strand break repair in ESCC cells. RAD18 upregulated p‐DNA‐dependent protein kinase complex (p‐DNA‐PKc) expression in vivo and in vitro.

**Conclusions:**

These data indicated that RAD18 may regulate radioresistance by facilitating NHEJ via phosphorylation of DNA‐PKcs in ESCC cells, providing a novel radiotherapy target for ESCC.

## Graphical abstract

RAD18 confer esophageal squamous cell carcinoma cells radioresistance.

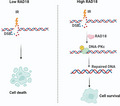

## 1 INTRODUCTION

Esophageal cancer (EC) is among the most prevalent malignancies and is reported to be the sixth major contributor to cancer‐associated fatalities globally. In East Asia, esophageal squamous cell carcinoma (ESCC) is the most commonly diagnosed histological type, accounting for over 90% of all EC cases.^1^, ^2^ Radiation‐based therapy, either by itself or in conjunction with chemotherapy, remains the standard treatment for unresectable ESCC. Unfortunately, ESCC cells easily develop radioresistance, and 31%–50% of patients with ESCC experience recurrence after radiotherapy (RT), which is associated with the unfavorable prognosis of patients with ESCC.^3^ Thus, there is an urgent need to investigate the molecular mechanisms underlying radioresistance in ESCC.

The biological function of ionizing radiation (IR) is to directly target DNA, which serves as the primary cellular target. The most severe and lethal types of DNA lesions caused by IR are DNA double‐strand breaks (DSBs), as a DSB alone is adequate to induce cell cycle arrest or trigger cell death. It is possible to repair DNA double‐strand breaks (DSBs) via two key pathways: homologous recombination (HR) and non‐homologous end‐joining (NHEJ).^4^ Although HR seems to be the most important process of repair in yeast, NHEJ may be the most important repair pathway in mammalian cells, particularly during the G1 phase of the cell division cycle.^5^ It is possible that the two broken ends of DNA are essentially patched together during NHEJ; however, this is only achieved in certain cases after minimal processing of the DNA ends, culminating in a fast recovery.^6^ The XRCC4–ligase IV complex putative DNA‐end processor enzymes, a protein kinase DNA‐dependent protein kinase complexes (DNA‐PKcs), and the DNA‐end binding Ku complex are required for NHEJ to occur.^7^ All these proteins have a role in maintaining the integrity of the mammalian genome. DBS repair following DNA damage is considered the primary process by which patients with ESCC are capable of resisting radiation treatment.

RAD18, an E3 ubiquitin‐protein ligase, is a modulator of DNA repair. It was first discovered in yeast, in which it participates in DNA DSB repair by interacting with RAD6.^8^ RAD18 has recently been discovered to be expressed aberrantly in a variety of malignancies, where it involves in the occurrence and progression of cancer.^9^, ^10^, ^11^ For instance, RAD18 can promote cervical cancer cell invasion by affecting the interleukin‐1β pathway.^12^ In addition, highly expressed RAD18 enhances the translesion synthesis and DSB repair, resulting in tolerance of replication stress and DNA damage, thus contributing to radioresistance.^13^ Our previous study indicated that RAD18 expression is positively associated with tumor invasion, metastatic recurrence, and unfavorable prognoses in patients with ESCC, and that this is attributable to Jun N‐terminal kinase (JNK)‐mediated activation of matrix metalloproteinase (MMP)‐2/MMP‐9.^14^ In the present study, we reported that the level of RAD18 expression in ESCC tissues is negatively related to the overall survival (OS) of patients receiving RT. RAD18 dramatically decreased ESCC cell radiosensitivity and facilitated NHEJ repair through the upregulation of p‐DNA‐PKcs.

## 2 MATERIALS AND METHODS

### 2.1 Obtaining tissue samples from patients

ESCC tissue samples from 108 patients who diagnosed with ESCC and received RT followed by surgery were acquired from the Nanjing Medical University Affiliated Suzhou Hospital (Jiangsu, China) between 2005 and 2009 (Table S1). Paraffin was used to embed all tissue samples. All the patients provided written informed consent before participating in the study. This study was approved by the Ethics Committee of Nanjing Medical University Institutional (number: KL901200).

### 2.2 Cell culture and irradiation

The Shanghai Institute of Cell Research (Shanghai, China) provided the human ESCC cell lines ECA‐109 and TE‐10. Short tandem repeat (STR) profiling was used to assess and validate the two cell lines. Cell lines stably expressing RAD18 were maintained in our laboratory.^14^ ECA‐109 and TE‐10 cells were cultured in Dulbecco's Modified Eagle Medium and Roswell Park Memorial Institute Medium‐1640 (Hyclone, USA) with 10% fetal bovine serum (Biological Industries, Kibbutz Beit HaEmek, Israel) at 37°C with 5% CO_2_. Radiation was administered at a dosage rate of 2 Gy/min and a peak energy of 6 MeV to the cells and tumor xenografts in nude mice using a linear accelerator (Varian).

### 2.3 Immunohistochemical analyses

The details of immunohistochemical (IHC) staining have been described in our earlier study.^14^Briefly, slices of tumor tissue were subjected to deparaffinization in xylene, followed by rehydration in ethyl alcohol at increasing concentrations. The slices were heated in a citrate buffer for 5 min for antigen retrieval. Non‐specific reactions were blocked for 30 min with 5% bovine serum albumin (BSA). Subsequently, the slices were incubated overnight with RAD18 antibody (MA5‐32284, Thermo Fisher Scientific) or p‐DNA‐PKc antibody (ab124918, Abcam) at a dilution of 1:100. After washing three times, horseradish peroxidase (HRP)‐conjugated antibody was introduced into the sample for 1 h, followed by coloring with 3–3′‐diaminobenzidine. The slices were washed, counterstained with hematoxylin, intermittently submerged in a water bath containing traces of ammonia, and dehydrated. The stained slices were imaged and scored using a Leica microscope (Leica Corporation, Germany). RAD18 protein expression was evaluated by two pathologists. RAD18 expression levels were defined as high (score ≥ 6) and low (score < 6).

### 2.4 Hematoxylin and eosin staining

The slices were deparaffinized in xylene and immersed in filtered hematoxylin (Beyotime Biotechnology) for 5 min. The slices were rinsed under running tap water in a staining box until the water was no longer colored. Subsequently, the slices were differentiated using 5% acetic acid for 1 min and rinsed with water. After staining with eosin (Beyotime Biotechnology) for 1 min and rinsing with water, the slices were dehydrated in ascending alcohol solutions. The stained slices were imaged using a Leica microscope (Leica Corporation).

### 2.5 RNA extraction and quantitative real‐time polymerase chain reaction

Total RNA was isolated using an RNA easy isolation reagent (Vazyme) according to the manufacturer's instructions. RNA concentrations were measured using NanoDrop 2000 (Thermo Fisher). Total RNA (1 μg) was transcribed into first‐strand cDNA using the HiScript III RT SuperMix (Vazyme). Real‐time polymerase chain reaction (PCR) was conducted using SYBR qPCR Master Mix (Vazyme) and a StepOne Plus instrument (Applied Biosystems, USA). The primers for human RAD18 and β‐actin were as follows: RAD18, forward: 5’‐GTCCTTTCATCCTCTACTCTCGT‐3′, and reverse: 5’‐TAGCCTTCTCTAGTTGTCTATCCC‐3′; and β‐actin, forward: 5’‐AGCGAGCATCCCCCAAAGTT‐3′, and reverse: 5’‐GGGCACGAAGGC‐TCATCACTT‐3′. The relative expression of RAD18 was calculated using the 2^−∆∆Ct^ method, and β‐actin was used as the internal control.

### 2.6 Colony formation assay

TE‐10 and ECA‐109 cells (200, 1000, 2000, 4000, 6000/well) were separately plated in six‐well plates for 24 h and then subjected to 0, 2, 4, 6, or 8 Gy X‐ray irradiation. Subsequently, the cells were cultured for 14 days, fixed with methanol, and stained with 0.5% crystal violet. Next, we tallied colonies containing over 50 cells. The surviving fraction of the experimental group was obtained by dividing the proportion of colonies in the experimental group by the proportion of colonies in the control group. The single‐hit multiple‐target radiobiological model, SF = 1 – 1 − exp(−*D*/*D*_0_)^*n*^, was used to fit the survival curves. The values of the sensitization enhancement ratio (SER) were calculated using the model.^15^ *D*_0_ is the mean lethal dose that represents an average for each cell to be hit once, and *D*_q_ is the threshold dose that represents the ability to repair non‐lethal injury.^16^

### 2.7 Immunofluorescence

TE‐10 and ECA‐109 cells were fixed with 4% paraformaldehyde and seeded on microscope slides in 24‐well plates for 10 min, followed by the addition of 0.2% Triton‐100 to permeabilize the cells. Additionally, non‐specific binding was blocked by the addition of 5% BSA for 1 h. Cells were then incubated at 4°C overnight with an anti‐γH2AX antibody (ab81299, Abcam). Cells were incubated once again with fluorescent secondary antibody (Multi Sciences) for 1 h and further stained with DAPI for 15 min. A ZEISS LSM800 (ZEISS) was used to determine the fluorescence of γH2AX in the samples.

### 2.8 Comet assay

TE‐10 and ECA‐109 cells treated with 4 Gy X‐ray radiation were subjected to DNA damage analysis using a neutral comet assay (Comet Assay Kit, Trevigen) according to the manufacturer's instructions. After rinsing with phosphate‐buffered saline (PBS), the cells were resuspended at a density of 5 × 10^5^ cells/ml, blended with molten LMAgarose at 37°C, and transferred onto a slide using a pipette immediately after mixing. SYBR Green I was used to stain the slides after they had been submerged in lysis buffer for electrophoresis and then dipped in the DNA precipitation solution. The Comet Assay IV platform (Perceptive Instruments) was used to determine the average tail moment for 50 cells per sample.

### 2.9 Homologous recombination and non‐homologous end‐joining assays

Liu et al. previously described a method for quantitatively assessing the activities of both NHEJ and HR using CRISPR/Cas9‐induced oligodeoxynucleotide (ODN)‐mediated DSB repair,^17^ which was used in this study. Briefly, HEK293T cells were seeded into six‐well plates at a density of 3 × 10^5^ cells/well. Following transfection with siRNAs, the Mre11‐Rad50‐Nbs1 complex inhibitor, mirin (50 μM), was used to examine the suppressive effects of HR activity. The inhibitory effects on NHEJ activity were investigated using the selective DNA‐dependent protein kinase inhibitor NU7441 (1.0 μM). Subsequently, 24 h later, ssODN or dsODN plasmids were transfected using Lipofectamine 3000 Reagent. To identify the marker sequence quantitatively, which acted as a surrogate indication of NHEJ and HR functions, PCR analysis was performed.

### 2.10 Western blot analysis

ECA‐109 and TE‐10 cells were collected and treated with radioimmunoprecipitation lysis solution (Beyotime Biotechnology) containing protease inhibitors for 15 min at 4°C. After isolation using sodium dodecyl‐sulfate polyacrylamide gel electrophoresis (10%, Millipore), the proteins were deposited onto poly(vinylidene fluoride) membranes. After blocking with 5% non‐fat milk, the membranes were incubated with primary antibodies against β‐actin (A82331134, Multi Sciences), RAD18 (MA5‐32284, Thermo Fisher Scientific), γ‐H2AX (ab81299, Abcam), Ku70/80 (ab166822, Abcam, UK), RAD51 (ab133534, Abcam), p‐DNA‐PKcs (ab124918, Abcam), and DNA‐PKcs (ab32566, Abcam) at 4°C overnight. Subsequently, the membranes were incubated with HRP‐conjugated anti‐rabbit or anti‐mouse secondary antibodies (Beyotime Biotechnology). Enhanced chemiluminescence (Beyotime Biotechnology) was used to visualize the protein bands. β‐actin was used as an internal control.

### 2.11 Experiments using tumor xenografts

Shanghai SLAC Laboratory Animal Co., Ltd. provided us with 5‐week‐old female BALB/c nude mice that were kept in pathogen‐free settings throughout the study process. Nude mice were randomly divided into four groups (ECA‐109 RAD18 and ECA‐109 NC, with or without IR). ECA‐109 cells were resuspended in PBS at a density of 2 × 10^7^ cells/ml. Nude mice received a subcutaneous injection of 0.2 ml of cell solution into the right groin of their bodies. Every 2 days, the tumors were examined using Vernier calipers to determine their size. The volume of tumor tissues was measured using the following equation: tumor volume (*V* mm^3^) = length (mm) × width^2^ (mm^2^)/2. Once the tumors reached a volume of 200 mm^3^, mice were subjected to X‐ray treatment at a dose of 8 Gy. Two weeks later, the tumors were collected for molecular analyses and evaluated using hematoxylin and eosin staining. The experimental protocols in this study were approved by the Animal Ethics Committee of Nanjing Medical University according to the guidelines of the Nanjing Medical University Animal Care and Use Committee.

### 2.12 Statistical analyses

The Statistical Package for the Social Sciences software (version 21.0) (IBM) was used to perform statistical analyses using Student's *t*‐test and one‐way analysis of variance. The results are expressed as the mean ± standard error of the mean from a minimum of three experimental replicates. A *p*‐value of less than 0.05 was considered statistically significant in the present study.

## 3 RESULTS

### 3.1 High RAD18 expression correlates with unfavorable prognosis of patients with esophageal squamous cell carcinoma receiving radiotherapy

**FIGURE 1:**
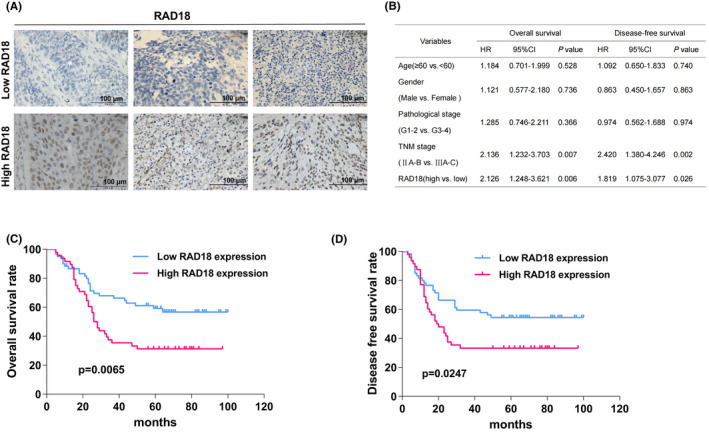
Elevated RAD18 expression in esophageal squamous cell carcinoma (ESCC) is associated with radiotherapy sensitivity and patient survival. (A) The expression of RAD18 in 108 ESCC tissue samples was investigated using immunohistochemical analysis. (B) Multivariate Cox analysis of factors related to the survival of patients with ESCC. (C,D) Survival analysis compared by two‐way analysis of variance illustrated that elevated RAD18 expression was correlated with a shortened disease‐free survival (*p* = 0.0247) and overall survival (*p* = 0.0065) in patients with ESCC receiving radiation therapy

In a previous study, we found that RAD18 was upregulated in ESCC and promoted the invasion of ESCC cells by regulating the JNK‐MMP pathway.^14^ To investigate the possible role of RAD18 in ESCC responsiveness to RT, we performed IHC staining to stain RAD18 expression in ESCC tissues obtained from 108 patients who had undergone RT. The characteristics of the 108 patients with ESCC are shown in Table S1. When comparing patients with elevated RAD18 expression levels (IHC score ≥ 6) to those with low RAD18 expression levels (IHC score < 6) (Figure 1A), survival analysis demonstrated that patients with higher RAD18 expression levels experienced a substantially shortened OS period (*p* = 0.0065) and disease‐free survival period (*p* = 0.0247) (Figure 1B–D). These data indicate that the levels of RAD18 protein are positively correlated with radioresistance and unfavorable prognosis of patients with ESCC receiving RT.

### 3.2 Downregulation of RAD18 boosts the sensitivity of ESCC cells to radiosensitivity

**FIGURE 2:**
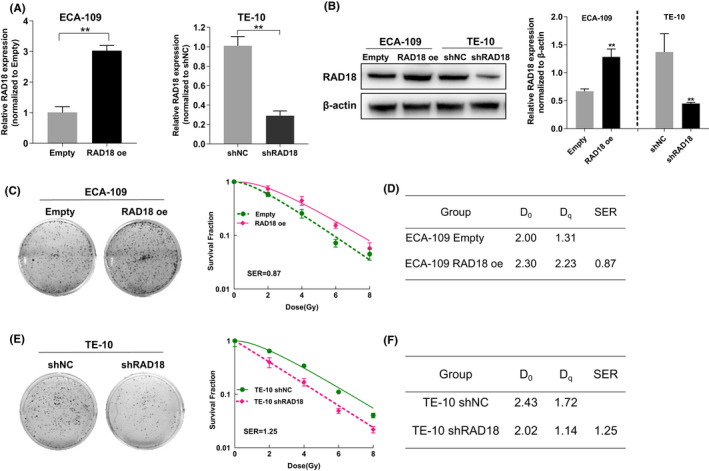
Suppression of RAD18 enhances radiosensitivity in esophageal squamous cell carcinoma (ESCC). (A) Quantitative polymerase chain reaction analysis of the knockdown or overexpression efficiency of RAD18 in TE‐10 cells and ECA‐109 cells. (B) Western blot analysis was utilized to evaluate the knockdown and overexpression efficiency of RAD18 in TE‐10 cells and ECA‐109 cells. (C,D) ECA‐109 cells were treated to 2, 4, 6, or 8 Gy of X‐ray radiation, and survival curves were plotted for the exposures. (E,F) TE‐10 cells were treated with 2, 4, 6, or 8 Gy of X‐ray radiation, and survival curves were plotted for the exposures. **p < 0.01

To further determine whether RAD18 is involved in radioresistance in ESCC cells, we established RAD18 overexpressed and control ECA‐109 cells and knocked down RAD18 expression in TE‐10 cells using shRNA (Figure 2A,B). The cells were then subjected to X‐ray irradiation at various doses. We found that RAD18‐overexpressed cells reserved stronger colony formation capability in contrast with that of control cells (SER = 0.87, Figure 2C,D). In contrast, the RAD18 downregulated cells showed a substantial decrease in colony formation capacity (SER = 1.25, Figure 2E,F). These data indicate that high RAD18 expression might contribute to the low radiosensitivity of ESCC cells to RT.

### 3.3 RAD18 increases double‐strand break repair capacity in ESCC cells

**FIGURE 3:**
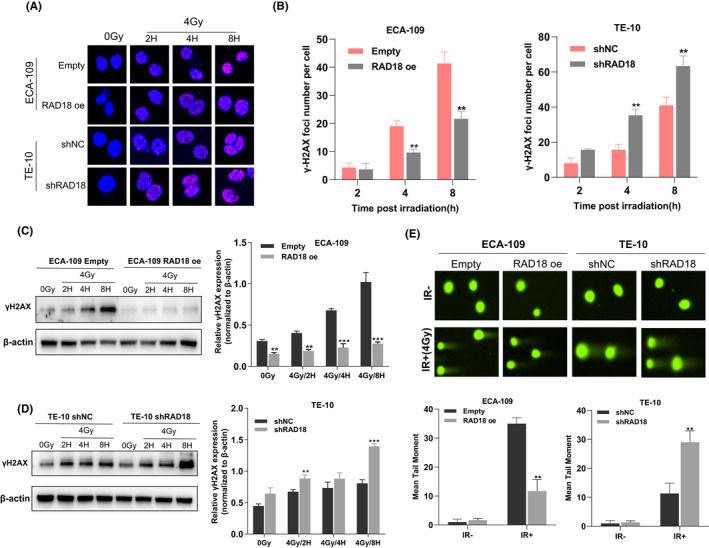
RAD18 increases DNA double‐strand break (DSB) repair capacity in esophageal squamous cell carcinoma (ESCC). (A,B) Immunofluorescence staining was performed to evaluate the γ‐H2AX foci in ESCC cells following RAD18 overexpression or knockdown. The foci numbers per cell were measured. (C) Western blotting analysis of the expression of γ‐H2AX in ECA109 cells at different time points treated with 4 Gy irradiation (IR). (D) Western blot analysis of the expression of γ‐H2AX in TE‐10 cells at different time points treated with 4 Gy IR. (E) Detection of DNA DSB damage following 4 Gy of IR was performed utilizing the neutral single‐cell gel electrophoresis test (comet assay). *p < 0.05, **p < 0.01, ^***^ *p* < 0.00

The DNA damage response (DDR) performs an integral function in the radiosensitivity of cancer cells with radiation treatment, and phosphorylation of H2AX on Ser139 (γH2AX) around DNA DSBs is a sensitive molecular marker of DDR. Here, immunofluorescence staining showed that RAD18‐deficient ESCC cells had more γH2AX foci per cell than control cells, whereas RAD18 overexpressed ESCC cells had fewer γH2AX foci per cell after 4 Gy of IR (Figure 3A,B). Similar results were obtained by western blot analysis of γ‐H2AX expression (Figure 3C,D). Additionally, a neutral single‐cell gel electrophoresis test (comet assay) was used to determine DBS damage 24 h following 4 Gy of IR. The comet tails of RAD18‐deficient ESCC cells were significantly longer than those of control cells, whereas ESCC cells overexpressing RAD18 had shorter comet tails than control cells (Figure 3E). These findings indicate that RAD18 can increase the DSB repair capacity in ESCC cells.

### 3.4 RAD18 regulates DSB repair capacity in ESCC cells by increasing p‐DNAPKcs expression after radiotherapy

**FIGURE 4:**
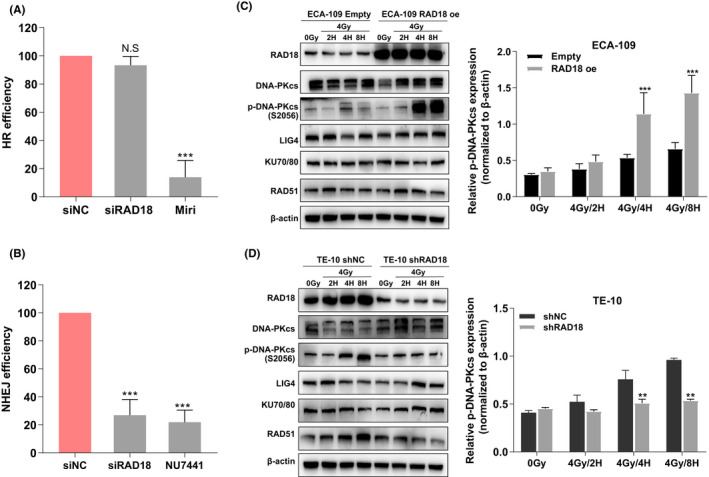
RAD18 facilitates non‐homologous end‐joining (NHEJ) and enhances phosphorylation of DNA‐PKcs. (A, B) CRISPR/Cas9‐induced oligodeoxynucleotide (ODN) detecting system was applied to monitor the efficiencies of NHEJ and HR. Mirin, an inhibitor of HR pathway; and NU7441, an inhibitor of NHEJ pathway. (C, D) Western blotting analysis of RAD18, DNA‐PKc, p‐DNA‐PKc (S2056), LIG4, Ku70/80, and RAD51 expressions in ECA109 and TE‐10 following RAD18 overexpression or downregulation at distinct time points following 4 Gy irradiation. ^**^ *p* < 0.01, ^***^ *p* < 0.00

The repair of DSBs is accomplished mostly via the HR and NHEJ processes. In the present study, the CRISPR/Cas9‐induced ODN detection system was used to examine the efficiency of NHEJ and HR. The findings revealed that RAD18 knockdown attenuated NHEJ activity in ESCC cells, but it did not affect HR‐mediated DSB repair (Figure 4A,B). Since RAD18 could modulate NHEJ repair of DSBs, we further determined whether RAD18 could affect the expression of major regulators of the NHEJ pathway in ESCC cells. Overexpression of RAD18 upregulated p‐DNA‐PKc (p‐S2056) levels, whereas downregulation of RAD18 decreased p‐DNA‐PKc (p‐S2056) levels at 4 and 8 h after 4 Gy IR. However, the alteration of RAD18 did not affect the expression of DNA‐PKcs, LIG4, RAD51, or Ku70/80 (Figure 4C,D). These findings indicate that RAD18 promotes DSB repair in ESCC cells by increasing the phosphorylation of DNA‐PKcs.

### 3.5 RAD18 confers ESCC cells resistance to irradiation in vivo

**FIGURE 5:**
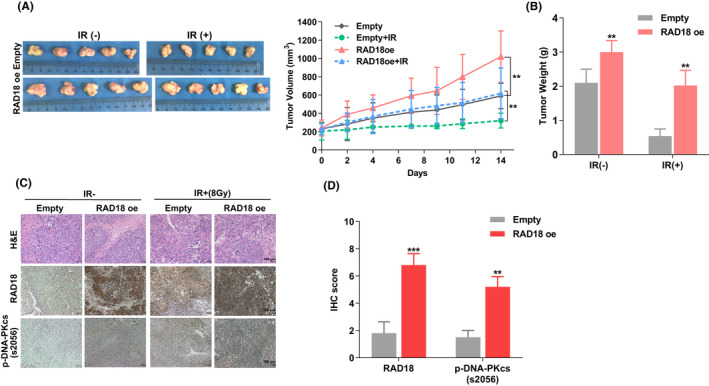
Resistance to irradiation in vivo conferred byRAD18 in esophageal squamous cell carcinoma. (A, B) The images and growth curve showed the volume from ECA‐109 cell‐derived (ECA‐109 NC and ECA‐109 RAD18) tumor xenografts in nude mice treated with radiation therapy. The tumor volumes were expressed as the mean ± standard error of the mean (SEM) of three separate experiments. (C, D) Hematoxylin and eosin(H&E) and immunohistochemical (IHC) staining analyses of RAD18 and p‐DNA‐PKcs (S2056) in the indicated tumor tissues from the xenografts. Counts or scores of IHC are expressed as the mean ± SEM. ^**^ *p* < 0.01, ^***^ *p* < 0.00

Nude mice bearing tumor xenografts were used to determine whether RAD18 induced radioresistance in ESCC cells in vivo. Upon reaching a tumor volume of 200 mm3, as shown in Figure 5A, mice were subjected to 6 MV X‐rays at a dose of 8 Gy (dosage rate, 2 Gy/min). Two weeks later, xenograft tumors were harvested. RAD18 overexpression contributed to an increase in tumor volume in contrast to the control group, and the tumors derived from RAD18 overexpressed cells showed less sensitivity to IR radiation compared to tumors formed by control cells (Figure 5A,B). Moreover, IHC analysis showed that RAD18‐overexpressed cells formed tumors with high levels of phosphorylated DNA‐PKcs, as opposed to tumors from the control group (p‐S2056) (Figure 5C,D).

## 4 DISCUSSION

IR is an essential element of routine treatment for patients with ESCC; however, developed radioresistance attenuates the therapeutic effectiveness of IR.^18^ As a result, identifying the factors that lead to IR resistance is crucial to the effectiveness of ESCC radiation treatment. In the present study, our results showed that high RAD18 expression was considerably correlated with the unfavorable prognosis of patients with ESCC receiving RT, which suggests that RAD18 may function as a regulator of IR resistance in ESCC cells. Numerous past studies have demonstrated that elevated levels of RAD18 gene expression are related to cancer progression and resistance to ionizing radiation in a number of malignancies.^11^, ^19^, ^20^ For example, RAD18 promotes cell proliferation in melanoma, and its elevated expression is correlated with patients' unfavorable survival.^21^ With respect to cervical cancer, RAD18 stimulates the migration as well as metastasis of carcinoma cells by activating the interleukin (IL)‐1 receptor pathway.^12^ Furthermore, it was shown that RAD18 might decrease apoptosis and increase cell proliferation in glioma cells, as well as facilitate resistance to radiation exposure.^13^, ^22^ Our previous study demonstrated that RAD18 facilitates tumor metastasis via the JNK‐MMP pathway in ESCC,^14^ and induces the resistance to RT and chemotherapy in colorectal cancer cells.^23^

In the present research, ESCC cells were found to be more sensitive to radiation when RAD18 was suppressed; however, RAD18 overexpression resulted in a converse effect on ESCC cell lines and tumor xenografts. These results were in agreement with the growing body of research regarding the resilience of RAD18 to DNA‐damaging chemicals or irradiation.^24^, ^25^, ^26^ Furthermore, we found that RAD18 enhanced radiosensitivity and increased DNA DSBs repair capacity in ESCC cells. Homologous recombination (HR) and non‐homologous end‐joining (NHEJ) have been shown to be the two key DNA damage repair pathways. In human glioma cells, RAD18 was found to disrupt HR repair and mediate resistance to IR.^13^ Interestingly, our findings indicated that loss of RAD18 markedly decreased NHEJ activity, but it does not affect the HR activity. In addition to our findings, Kenji et al. reported that RAD18 facilitated 53BP1‐directed DSB repair by increasing the preservation of the 53BP1 protein.^24^ These findings indicated that RAD18 is involved in DSB repair via the NHEJ pathway.

The DNA‐dependent protein kinase complex (DNA‐PKcs), which is composed of the Ku70/80 heterodimer and the catalytic component DNA‐PKcs, is necessary for the synapsing of damaged DNA ends and the facilitation of repair of the DNA broken in the NHEJ process. DNA‐PKcs is the most important modulator of the NHEJ pathway because its kinase activity is necessary for NHEJ‐mediated DNA double‐strand break repair.^27^ When DNA‐PKcs is activated as a consequence of ionizing radiation or radiomimetic agents treatments, it phosphorylates at the S2056 and T2609 sites in a short period of time.^28^ Since then, phosphorylation at the S2056 has extensively been utilized as the indicator for DNA‐PKcs activation.^29^ Moreover, human replication factor C4 is identified as another important regulator by genome‐wide RNAi screen in colorectal cancer, which regulates NHEJ pathway to cause radioresistance by interacting with Ku70/Ku80.^30^ In the present research, our data illustrated that over‐expressed RAD18 substantially elevated the p‐DNA‐PKcs (S2056) expression in ESCC cells after 4 Gy IR, whereas depletion of RAD18 contributed to a substantial reduction in p‐DNA‐PKcs (S2056) expression. Moreover, RAD18 has a positive correlation with the p‐DNA‐PKcs (S2056) expression in vivo as well. However, RAD18 could not affect the DNA‐PKcs, LIG4, RAD51 expression, and Ku70/80. In addition, highly expressed RAD18 induces radioresistance via downregulating the P53 expression and facilitating its nuclear export in glioma.^19^ Our findings indicated that RAD18 may regulate radioresistance by facilitating NHEJ via phosphorylation of DNA‐PKcs in ESCC cells.

In conclusion, we firstly revealed that RAD18 contributed to ESCC cell resistance to IR, which might be mediated by the NHEJ pathway. Our findings indicate that RAD18 may be a promising target for improving the sensitivity of patients with ESCC to IR treatment. In addition, RAB18 inhibitors may be a new and effective treatment for tumors with DNA repair deficiencies. However, our study also has a limitation in that the underlying molecular mechanism of RAD18‐mediated phosphorylation of DNA‐PKcs and how RAD18 promotes NHEJ remains unclear and needs to be further explored in the future.

## CONFLICT OF INTEREST

There is no potential conflicting interest to disclose.

## AUTHOR CONTRIBUTIONS

SHW, YHC, and JDZ designed and supervised the study. XQL, STZ, and LSZ conducted the experiment. ADG and JX contributed to the acquisition of results. HC analyzed the data. XQL and JDZ wrote the manuscript. All authors have read and approved the final version of the manuscript.

## ETHICS STATEMENT

Written informed consent was provided by all the patients in The Affiliated Suzhou Hospital of the Nanjing Medical University for the use of ESCC and adjacent tissue sample in the present study. The Institutional Ethics Committee of Nanjing Medical University granted its approval for the present research.

## Supporting information


Table S1

## Data Availability

The authors will provide data included in the present research upon request.
